# A molecular propeller effect for chiral separation and analysis

**DOI:** 10.1038/ncomms8868

**Published:** 2015-07-28

**Authors:** Jonathon B. Clemens, Osman Kibar, Mirianas Chachisvilis

**Affiliations:** 1 Dynamic Connections, LLC, 6150 Lusk Boulevard B104, San Diego, California 92121, USA

## Abstract

Enantiomers share nearly identical physical properties but have different chiral geometries, making their identification and separation difficult. Here we show that when exposed to a rotating electric field, the left- and right-handed chiral molecules rotate with the field and act as microscopic propellers; moreover, owing to their opposite handedness, they propel along the axis of field rotation in opposite directions. We introduce a new molecular parameter called hydrodynamic chirality to characterize the coupling of rotational motion of a chiral molecule into its translational motion and quantify the direction and velocity of such motion. We demonstrate >80% enrichment level of counterpart enantiomers in solution without using chiral selectors or circularly polarized light. We expect our results to have an impact on multiple applications in drug discovery, analytical and chiral chemistry, including determination of absolute configuration, as well as in influencing the understanding of artificial and natural molecular systems where rotational motion of the molecules is involved.

## Abstract (web summary)

Separation and analysis of enantiomers is a difficult task due to the normally identical physical properties they display. Here, the authors show how chiral molecules with non-zero dipoles can be propelled in opposite directions under the influence of a rotating electric field.

## Graphical abstract

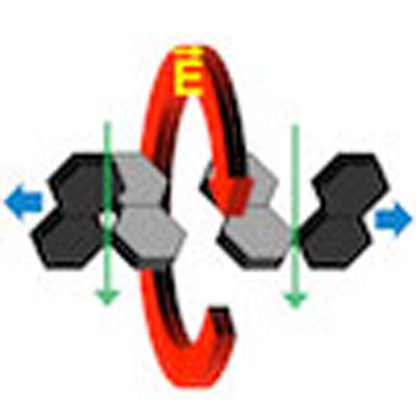

The relevance of chirality in Nature is well established^1^. A variety of mechanisms have been proposed to explain symmetry-breaking interactions and the origins of enantiomeric homogeneity in biological systems, such as circularly polarized light^2^, gravitational fields and vortex motion, parity violation, time-dependent optical and magnetic fields or photochemistry^3^. These mechanisms mostly lead to very small enantiomeric excess (ee) and thus require additional amplification to reach an enantiopure state.

Separation and analysis of chiral molecules also plays an important role in the pharmaceutical industry^4^. Current separation methods typically rely on interactions with various chiral selectors, for example, chiral chromatography or recrystallization and related Viedma ripening^5^,^6^, while determination of absolute configuration relies on X-ray crystallography and on chiroptical spectroscpy^7^, which encompasses a range of spectroscopic techniques, including vibrational circular dichroism^8^. All these methods are time consuming and they do not lend themselves to *a priori* predictions of performance for newly synthesized molecules. Other recently proposed chiral separation/analysis methods include chiral gratings^9^ and NMR in the presence of static electric field^10^,^11^. Very recently, molecular handedness has been detected using microwaves^12^ and Coulomb explosion imaging^13^; however, these new methods are applicable to molecules that can be sampled in the gas phase and have not been demonstrated for large molecules with high degrees of conformational freedom.

Pasteur^14^ claimed that dissymmetry generated by a rotation coupled with linear motion is similar to spatial dissymmetry (chirality) as encountered in chemical structures^1^. In addition, the macroscopic propeller effect is a well-known hydrodynamic phenomenon that manifests itself through rotational–translational coupling in left–right dissymmetrical bodies such as helical filaments^15^,^16^,^17^. Thus, chiral molecules can also be envisaged as tiny propellers with their ‘handedness' and propulsion direction being determined by absolute configuration of the molecule. It has long been hypothesized by Baranova *et al.*^18^, based on phenomenological theory, that the molecular ‘propeller effect' may lead to separation of enantiomers exposed to radiofrequency electric fields of rotating polarization. However, owing to deficiencies in the theoretical derivations, the molecular propeller effect was predicted to be rather small, which may have discouraged experimental verification of the existence of such an effect. There have been a few experimental studies on separation of macroscopic chiral objects (such as helical colloidal particles, model chiral helices, helical-shaped bacteria, >1 μ) in helical flows, vortices, microfluidic shear flows and rotating magnetic fields^19^,^20^,^21^,^22^; however, overcoming Brownian diffusion that starts to dominate on nanoscale has been a challenge until now.

Here we show both theoretically and experimentally that the molecular propeller effect with rotating electric fields (REF) offers an important new chiral separation and analysis method. We first discuss the relationship between absolute configuration and handedness of a chiral molecule and its propulsion direction followed by the analysis of molecular dynamics simulations used for quantitative determination of rotational–translational coupling. After deriving the theoretical expression for the molecular propeller effect, we discuss experimental results. Finally, we mention how the molecular propeller effect can have an impact on the field of chiral separations and analysis.

## Results

### Rotational–translational coupling

For demonstration purposes we selected two different binaphthyl molecules because of their apparent similarity to a two-blade propeller and suitable orientation of their dipole moments (see Methods section, ‘Reagents'). When such a chiral molecule in solution is exposed to the REF, its dipole moment will tend to reorient and align with the electric field direction, leading to rotation of the molecule. With the macroscopic propeller analogy in mind, opposite enantiomers will propel in opposite directions along the chamber, leading to separation. Such an effect also offers a means of determining absolute configuration, provided that the direction of propulsion can be predicted theoretically.

**Figure 1:**
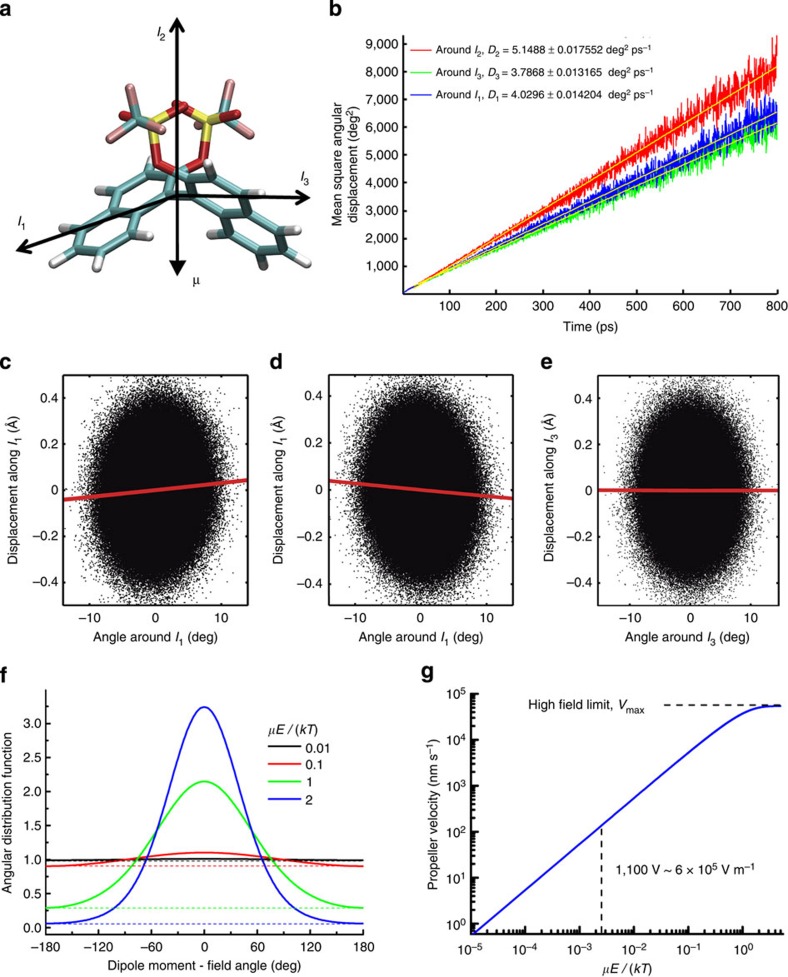
Theoretical results. (**a**) Binapthyl molecule I (S enantiomer). Dipole moment, *μ*=5.3 Debye. (**b**) The mean square angular displacement of molecule I around three different molecular rotation axes. (**c**–**e**) Displacement of the centre of mass of the molecule along a specified axis versus the angle of rotation around the same axis (only part of the trajectory is shown to reduce the number of points on the graph). Each data point represents a time step of 250 fs. (**c**,**e**) S enantiomer. (**d**) R enantiomer. Red lines indicate linear regression fit to the data. Slope values (**c**) 1.22±0.03 Å, (**d**) −1.18±0.03 Å, (**e**) −0.02±0.03 Å per 360 deg revolution (mean±s.e.m.). (**f**) Distribution of molecules as a function of the angle between the external electric field and the dipole moment of the molecule (*α*), plotted for four different values of dipole moment—electric field interaction energies. Molecules above the dashed line represent the ‘responding' fraction of the molecules that respond to changes in electric field direction. (**g**) The dependence of the expected propeller velocity on relative electric field magnitude calculated according to equation (4) for the following parameters: molecule rotation frequency, *ν*_eff_=0.9 MHz, displacement per one revolution, *L*_rev_=1.2 Å.

We introduce hydrodynamic chirality to characterize the direction and magnitude of propeller-like motion of a molecule. The dipole moments of the selected binaphthyl molecules are parallel to the C_2_ symmetry axes (which is aligned along the I_2_ axis in Fig. 1a). An applied external electric field will impose a torque around any axis in the coordinate plane containing I_1_ and I_3_ axes (Fig. 1a) depending on the spatial orientation of the molecule. On the basis of symmetry considerations, it is expected that rotation around the I_1_ axis will propel the molecule because of the propeller effect, while rotation around the I_3_ axis (aligned along internaphthyl bond) should not lead to significant propulsion. Owing to random orientations of the molecules in solution with respect to the plane of the REF, the molecule on average will rotate around all possible axes in the I_1_ and I_3_ planes. On the basis of a simple hydrodynamic picture, the propulsion direction for a rotating binaphthyl critically depends on the dihedral angle between naphthyl moieties—similar to propeller blades. Therefore, the propulsion direction is expected to be of the opposite sign for different absolute configurations S and R.

To characterize rotational–translation coupling and estimate the expected amount of propulsion per one revolution of the molecule, we have performed molecular dynamics simulations with the explicit solvent (benzene) (Methods). Random rotational motions (rotational diffusion) due to thermal torques acting on the molecule around selected molecular axes (Fig. 1b) were used to correlate the amount of rotation of the molecule around the selected axis with the amount of displacement along the same axis (Fig. 1c–e). Figure 1c,d indicates that there is a correlation between rotation around axis I_1_ and the propulsion along this axis; moreover, the direction of propulsion is opposite for the S and R enantiomers. As expected, Fig. 1e shows that there is no propulsion when the molecule rotates around the I_3_ axis. A linear fit to the data similar to those shown in Fig. 1c,d yields the propeller efficiency, that is, the value of displacement per one revolution (*L*_rev_) of 1.22±0.03 and −1.18±0.03 Å per revolution for the S and R enantiomers of Molecule I, respectively. In contrast, *L*_rev_=−0.02±0.03 Å for rotation around the I_3_ axis. Similar MD simulations performed for molecule II yield *L*_rev_ values of only 0.19±0.03 and −0.17±0.03 Å (for S and R configurations, respectively). This could be rationalized by the presence of an additional linker chain between the naphthyls that presents a deviation from an ideal propeller shape.

### Theoretical analysis of molecular propeller effect

Next, we derive the dependence of the molecular propeller effect on electric field magnitude (*E*), rotation frequency (*ν*) and electrical dipole moment of the molecule (*μ*). We assume that electric field rotation is much slower than the rotational relaxation time of the molecules (in low-viscosity solvents, typically in the 100–500-ps timescale^23^,^24^). Under this assumption, inertial effects can be neglected and the angular distribution density function, *ρ*(*ϕ*, *θ*, *t*), is given by the solution of the Smoluchowski equation in spherical coordinates^25^:


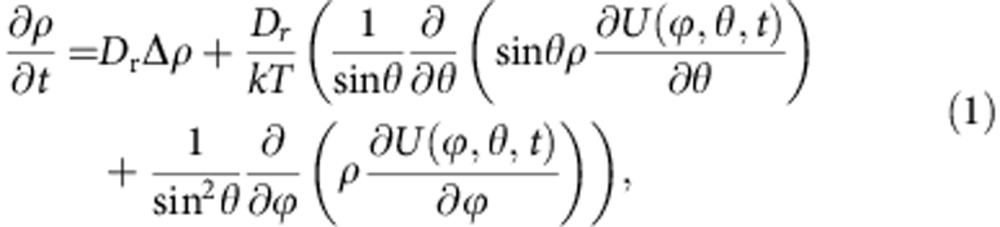


where 
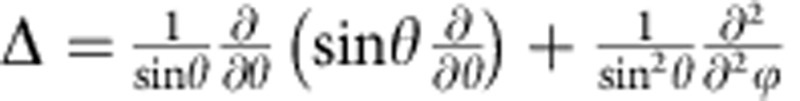
 is the angular Laplace operator, *U* (*ϕ*, *θ*, *t*)=−**μ**(*ϕ*, *θ*, *t*)·**E**(*ϕ*, *θ*, *t*) is the dipolar interaction energy (**μ** and **E** are dipole moment and electric field vectors, respectively), *D*_r_ is the rotational diffusion coefficient, *k* is the Boltzmann constant and *T* is temperature. When the electric field rotates slowly, the distribution of molecules in angular space can be assumed to be always at equilibrium with any electric field orientation. The equilibrium solution of equation (1) is in the form of a simple Boltzmann distribution:


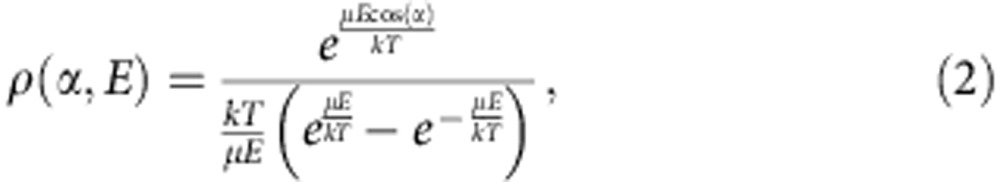


where *α* in the relative angle between the electric field vector and dipole moment; *μ* and *E* are the absolute values of dipole moment and electric field magnitude, respectively (since distribution is axially symmetric around the direction of electric field, there is no dependence on the azimuthal angle *ϕ*; see Methods for derivation). This bell-shaped function exhibits a peak at an angle corresponding to the electric field orientation; Fig. 1f shows a two-dimensional projection of this function (for a time instant when electric field is parallel to the *z* axis, this could be any plane containing the *z* axis—see Supplementary Fig. 1). When the electric field rotates, the peak of the distribution function follows the electric field vector; however, its shape does not change, provided molecules have sufficient time to adapt to the new direction of the field. Then, the ‘responding' fraction of molecules (*F(E)*) can be calculated as a ratio of molecules that follow the rotation of the electric field (that is, all molecules above the dashed lines in Fig. 1f) to the total number of molecules:


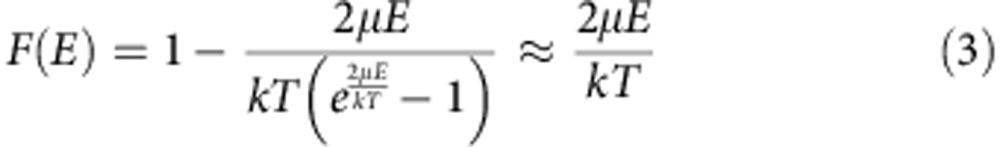


(see Methods, Calculation of the Responding Fraction. Assuming an electric field magnitude of 6 × 10^5 ^V m^−1^ and a molecular electric dipole moment of 5.3 Debye (molecule I, see Methods), *F(E)*∼5 × 10^−3^ at room temperature, that is, on average only 5 molecules out of 1,000 are rotating with the REF at any given time. However, it should be noted that all molecules rotate, both because of random thermal torques and the effect of the REF. However, the molecules belonging to a fraction below the dashed line (Fig. 1f) rotate equally randomly in both directions (as if they do not ‘experience' the electric field torque) and therefore they do not contribute to propeller propulsion. Whereas the fraction of molecules above the dashed line (a ‘responding' fraction) will rotate following the electric field rotation (in other words, the peak of the angular distribution will follow the electric field orientation) resulting in directional propeller motion mediated by rotational–translational coupling for those molecules. Alternative interpretation of *F(E)* could be that all molecules rotate but at a reduced, average frequency; for example, if field rotation frequency is 0.9 MHz, then all molecules rotate at 4.5 kHz (that is, 0.9 MHz × *F*(*E*)). Note, however, that this is not the effective rotational frequency we introduce below as there is an additional dynamic effect of molecules lagging if electric field rotational frequency is higher than molecules can follow (see Methods, Effective Rotational Frequency).

The velocity of a molecule owing to the propeller motion can be expressed as:


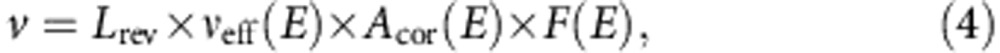


where *ν*_eff_ is an effective molecular rotation frequency and *A*_cor_ is angular correction factor. Excluding the *A*_cor_(*E*) × *F*(*E*) factor, the equation (4) simply means that linear propulsion velocity is due to rotational-to-translational coupling. As indicated above, *F*(*E*) accounts for the fact that only a small fraction of molecules follow the REF, while the factor *A*_cor_ accounts for the random orientation of the propeller axis; *A*_cor_≈0.5 for parameters used in this paper (see Methods, Calculation of Angular Correction Factor *A*_cor_).

Figure 1g shows that for weak electric fields, propulsion velocity is linearly proportional to rotation frequency and field magnitude (only in the case if field rotates slower than *ν*_esc_). In addition, note that the linear dependence of propeller velocity on REF frequency (equation (4)) suggests that increasing frequency can more than compensate for the low *F(E)*, enabling achievement of high propulsion velocities and separation and/or analysis within a short time period. However, in equation (4), *ν*_eff_ is equal to the rotation frequency of the REF only in the case when the molecular population is able to respond to a change in the orientation of the external electric field sufficiently fast, that is, it is able to follow the REF. The responding fraction of the molecules will be able to rotate at the frequency of the external REF if the maximal torque imposed by the dipole–field interaction is larger than the rotational drag torque because of the rotational friction experienced by the molecule in solution at the REF frequency. Above a certain REF frequency, the molecules are not able to follow the REF (see Methods and Supplementary Fig. 2); the value of this ‘escape' frequency, *ν*_esc_, is determined by rotational friction, the dipole moment and electric field strength. More specifically, *ν*_esc_ is a frequency at which the torque due to rotational drag is equal to the maximal torque imposed by the electric field on the molecular dipole: 
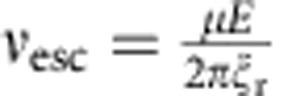
 where 
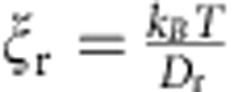
 is the rotational friction coefficient. The *ν*_esc_ is ∼0.51 and ∼2.1 MHz for molecules I and II, respectively (see Methods). At REF frequencies above *ν*_esc_, the molecules ‘slip', rotating at greatly reduced effective frequency
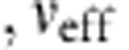
 (see Effective Rotational Frequency in Methods and Supplementary Fig. 2b). For example, for molecule I exposed to the REF of 0.9 MHz, *ν*_eff_ is only156 kHz; in this case equation (4) yields a velocity value of 25 nm s^−1^ at which molecule I is propelled in solution because of the propeller effect. The propulsion velocity of molecule II is larger at ∼45 nm s^−1^, mostly because of its much higher rotational escape frequency since *L*_rev_ for molecule II is significantly lower (for more details see Methods). The equation (4) is only strictly valid when *ν*_esc_ is significantly higher than the frequency of the REF (that is, when the assumption of equilibrium distribution (equation (2)) applies); nonetheless, our approximation based on introduction of *ν*_eff_ is supported by experiment (see below). Note that when the field rotation frequency is much higher than *ν*_esc_, *ν*_eff_∼*E*^2^ (see equation (9) in Methods); it then follows from equation (4) that the overall propeller velocity is proportional to the cube of the electric field magnitude. For this reason our set-up was designed to operate in the regime when the dependence on the electric field is nearly linear, that is, at higher voltage rather than higher frequency.

### Experimental demonstration of molecular propeller effect

**Figure 2:**
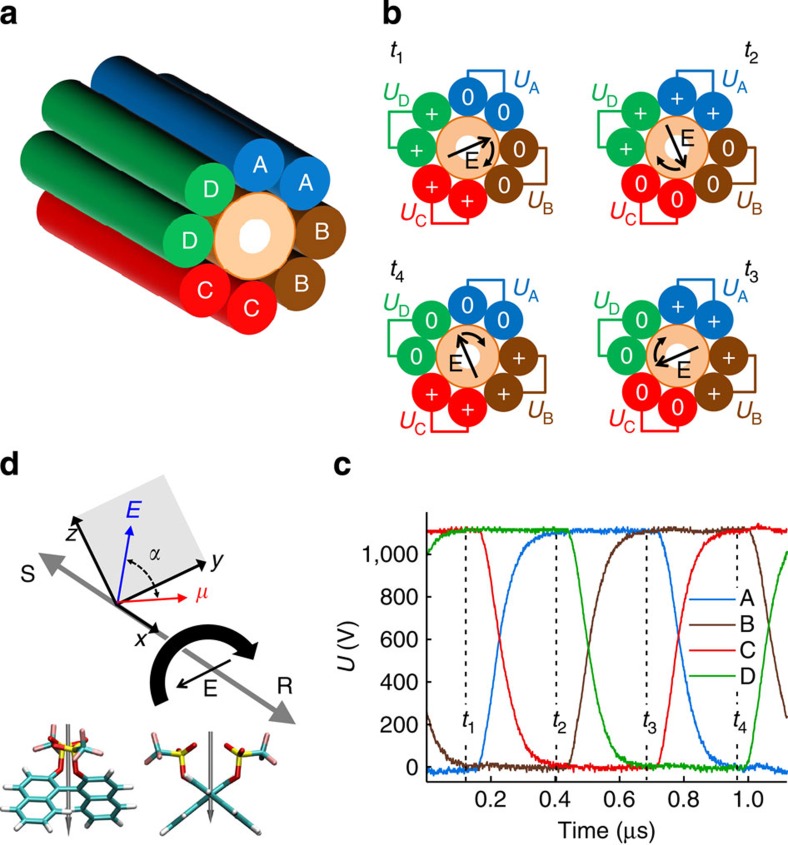
Experimental set-up. (**a**) A three-dimensional slice of the separation chamber showing the four electrodes, A–D, surrounding the microfluidic capillary. (**b**) Cross-section schematic showing how the electric field rotates within the separation chamber at four selected time points during a single cycle, *t*_1_ to *t*_4_. At each time point, two electrodes are in the high-voltage state (+), and the opposite two electrodes are in a zero-voltage state (0). This results in a 90° rotation of the orientation of the electric field (E) within the separation chamber between each time point. (**c**) The voltage waveforms on each of the four electrode pairs during one full cycle of the electric field rotation. These square-like waveforms (1,100 V, 900 kHz) show the *π*/2 phase shift between electrodes A–D. The four time points, *t*_1_ to *t*_4_ from **b** are also shown. (**d**) Expected directions of motion of the S and R enantiomers of molecule I for the indicated direction of rotation of the REF (CW, curved black arrow). *α* is the relative angle between the electric dipole moment and electric field. Electric field rotates around the *x* axis in the plane *zy*. The grey arrows show the (opposite) directions of motion for the S and R enantiomers of molecule I. The structure of molecule I (S enantiomer) is also shown with the dipole moment direction indicated.

**Figure 3:**
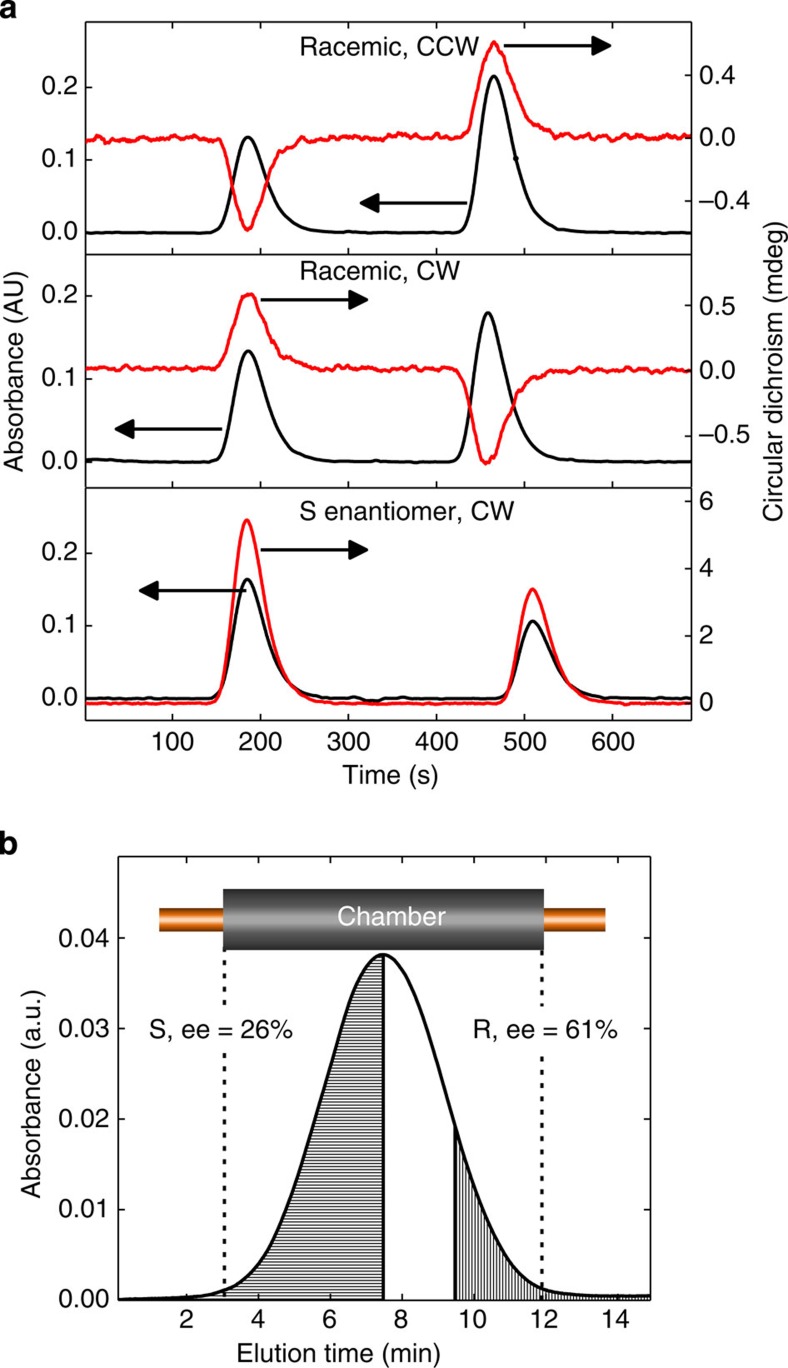
Experimental results for Molecule I. (**a**) Absorbance and CD chromatograms (obtained simultaneously) for samples of molecule I after being exposed to the REF for 83 h and subsequently collected. All process conditions were identical, except where noted. The first (left) peak of each chromatogram represents the leading half of the slug and the second (right) peak represents the trailing half of the slug. Upper: racemic molecule I after exposure to counterclockwise (CCW) REF. Middle: racemic molecule I after CW REF. Lower: pure S enantiomer of molecule I after CW REF. (**b**) Absorbance chromatogram from the in-line detector of a slug of racemic molecule I after exposure to CW REF for 45 h. The sample collected from the shaded left side of the chromatogram had ee of 26% of the S enantiomer of molecule I, while the right shaded section of the chromatogram had ee of 61% of the R enantiomer of molecule I. This is consistent with the edge of the sample slug being more enantiomerically enriched than in the centre. The boundaries of the active area of the separation chamber are also shown after conversion to the elution timescale.

To experimentally confirm the molecular propeller effect, we have performed experiments on solutions of molecules I and II using the experimental apparatus depicted in Fig. 2a. The REF inside the microfluidic chamber is generated by applying *π*/2 phase-shifted voltages to the four pairs of electrodes surrounding the chamber (Fig. 2b,c). A small amount of a racemic solution was injected into the centre of separation chamber and exposed to the REF. Data in Fig. 3a show that the materials collected from the leading and trailing sides of the exposed sample (which was split into two halves at the centre of the absorption chromatogram) have finite and opposite signs of circular dichroism (CD) signal. If the rotation direction of the REF is inverted, the CD signals from the leading and trailing fractions are inverted too. Furthermore, when the experiment is performed on a pure enantiomer sample, no inversion of the CD signal for leading and trailing fractions occurs; these results unequivocally prove that exposure to the REF leads to enantiomeric separation of binaphthyl molecules. Importantly, the experimentally detected direction of propulsion of S and R enantiomers is the same as predicted by MD simulations (compare signs of *L*_rev_ in Fig. 1c,d and CD signals in Fig. 3a and Supplementary Fig. 3a; for example, the leading fraction is enriched with the S enantiomer for the clockwise (CW) REF, see Fig. 3a middle panel). This offers a new approach for determining absolute configuration of a chiral molecule.

To quantify chiral separation efficiency we use ee (ee=0 % for racemic sample and ee=100% for pure enantiomer). Using a model of diffusive spreading of two overlapping Gaussian concentration profiles with opposite linear drift terms due to propeller effect (corresponding to the S and R enantiomers moving in the opposite directions), it is straightforward to show that the ee is given by:


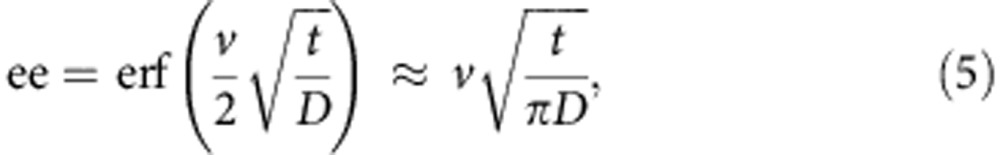


where *D* is the translational diffusion coefficient (equation (5) is derived assuming the sample is split into two halves at the centre of the absorption profile). Intuitively, a square root dependence is indeed expected for low enrichment levels since a linear drift term due to propeller effect (*vt*) is acting against the diffusive spreading of the molecules (
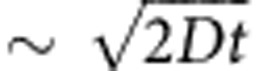
). On the basis of the experimentally determined translational diffusion coefficient (see Methods) and a drift term of 25 nm s^−1^, equation (5) predicts the ee value of 19%, which is reasonably close the experimental value of ∼26% (Fig. 3b) considering uncertainties in many parameters used to calculate *v*. Moreover, Fig. 3b indicates that enantiomeric enrichment is higher if the sample is collected from an off-centre location of the chromatographic absorption profile, as compared with the ee value from one full half of the sample (ee=61%, corresponding to 80.5% enrichment level); this is expected for a diffusive spreading process with the presence of a drift term.

**Figure 4:**
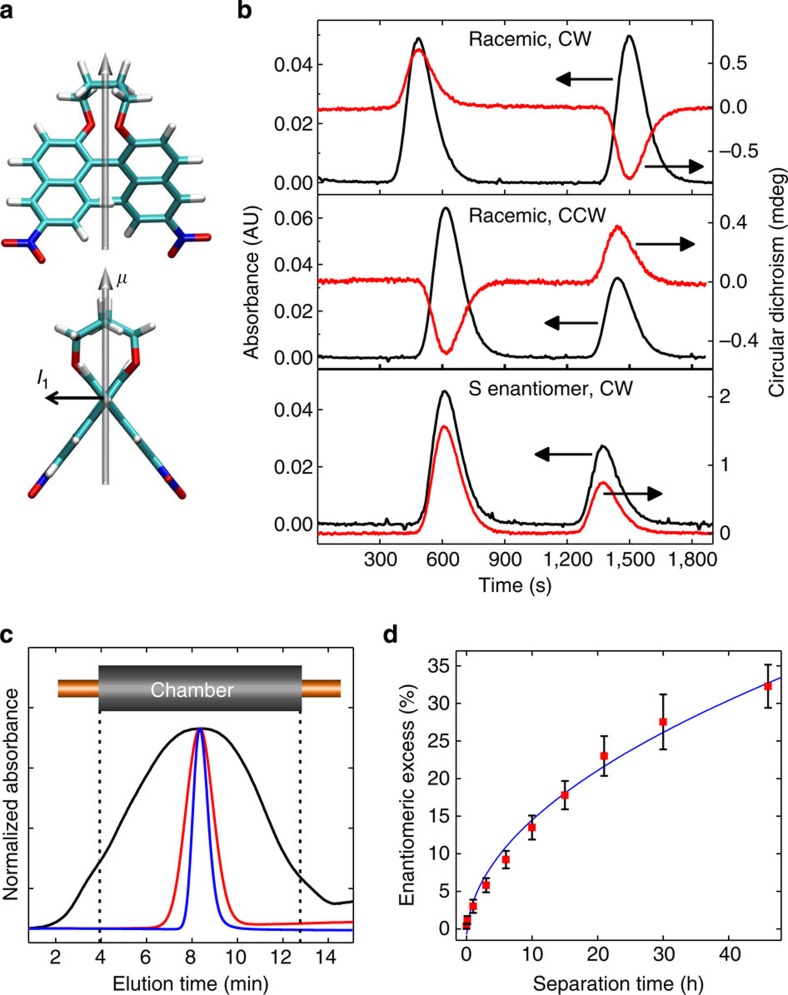
Experimental results for Molecule II. (**a**) Binaphthyl molecule II (S enantiomer). Dipole moment, *μ*=10.9 Debye. (**b**) Absorption and CD chromatograms (obtained simultaneously) for racemic molecule II, after being exposed to the REF for 21 h and subsequently collected. All experimental conditions were identical, except where noted. The first (left) peak of each chromatogram represents the signal from the leading half of the slug and the second (right) peak represents the trailing half of the slug. Upper panel: racemic molecule II after exposure to CW REF. Middle panel: racemic molecule II after exposure to CCW REF. Lower panel: pure S enantiomer of molecule II after exposure to CW REF. (**c**) Three overlaid and normalized absorbance chromatograms from the in-line detector of racemic molecule II after exposure to CW REF for 8 min (blue), 6 h (red) and 60 h (black). The separation chamber boundaries, converted to the elution timescale, are also shown. The black chromatogram shows shape distortions due to effects from the chamber boundaries. (**d**) ee versus CW REF exposure time for racemic molecule II. Each sample of molecule II was collected by splitting the sample at the centre of the in-line chromatographic absorption profile into the leading and trailing halves, with the leading half being enriched in the S enantiomer and the trailing half being enriched in the R enantiomer. Each data point is an average of at least six samples (both S and R ee values averaged together), with s.e. bars shown. The blue curve is the least squares fit of equation (5) to the data.

We further tested whether higher separation of enantiomers could be achieved using molecule II (Fig. 4a), which has a higher dipole moment (10.9 D). CD chromatograms in Fig. 4b clearly show that after exposure to the REF, the leading and trailing fractions of the initially racemic sample become chiraly enriched. In contrast, when the experiment is performed on a pure enantiomer sample, no inversion of the CD signal from leading and trailing fractions occurs. Moreover, and similar to molecule I, the sign of the CD signature depends on the direction of rotation of the REF, and the direction of translational motion is correctly predicted by MD simulation for S and R configurations, for example, the leading fraction is enriched in the S enantiomer after exposure to the CW REF (compare CD signs in Fig. 4b upper panel and Supplementary Fig. 3b). Figure 4c shows concentration profiles after various exposure times to the REF, while Fig. 4d summarizes ee values as a function of separation time. These data indicate that ee is already detectable after 1 h of exposure to the REF. The enrichment curve in Fig. 4d exhibits a square root time dependence, as predicted by equation (5). This confirms that the underlying separation mechanism is linear in time, as expected for the propeller motion. A fit of equation (5) to the ee data in Fig. 4d using the experimentally determined value of *D* (see Methods) results in a propulsion velocity of ∼50±5 nm s^−1^, which is very close to theoretically predicted value (45 nm s^−1^, see above). The theoretical enrichment estimate for molecule II is 27% after 46 h, which is also very close to the experimentally observed value of ∼32%. At higher exposure times, separation efficiency can deviate from the theoretical value primarily because of the finite length of the active area of the separation chamber (10 cm). The material that diffuses outside of the chamber boundaries is not covered by the electrodes, and therefore is not exposed to the REF.

## Discussion

Our theoretical findings on the molecular propeller effect are fundamentally different from an earlier theoretical study by Baranova *et al.*^18^, which predicted a quadratic dependence on electric field magnitude based on a phenomenological assumption that propeller velocity should be proportional to radiofrequency field intensity (∼*E*^2^). Moreover, they have assumed that a rate at which molecule settle in response to field change is faster than 100 MHz, which as we show above is not correct for typical small molecules (that is, they did not account for the fact that molecules would not be able to rotate at radiofrequencies). Using the field magnitude of 3 × 10^5 ^V m^−1^ and radio field rotation frequency of 100 MHz proposed in their study^18^, the *ν*_eff_ would be only 308 Hz for molecule I, making experimental observation of the effect not practically feasible). In contrast, our derivation of equation (4) is based on the solution of rotational diffusion equation with two degrees of freedom, is valid for any electric field magnitude and predicts that the propeller effect at practical electric field magnitudes will be multiple orders of magnitude stronger than predicted by Baranova *et al.*, making experimental verification and potential applications feasible. For example, if we use the theory proposed in the paper by Baranova *et al.* to calculate the propulsion velocity for molecule I in our experimental set-up, we obtain a value of only ∼0.3 nm s^−1^, which is about two orders of magnitude lower than that predicted by our approach.

As illustration if a solution of enantiomers is placed into a closed container of length *L* and exposed to an REF, over time an exponential concentration distribution will develop:


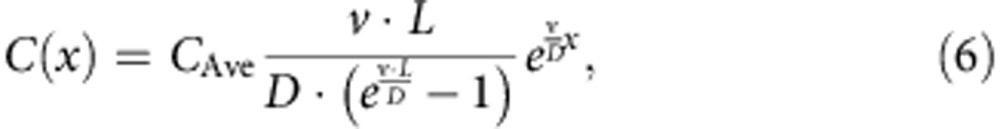


where *C*_Ave_ is the average concentration of enantiomers and *D* is the translation diffusion coefficient. The distribution profile is inverted for opposite enantiomers. Equation (6) follows directly from the lateral diffusion equation with a drift term due to propeller motion. Quantity *D*/*v* has a dimension of length and characterizes the balance between diffusion and drift motion due to the propeller effect. A smaller characteristic length indicates that the propeller effect is stronger than diffusion, allowing a more complete separation of enantiomers within a shorter time period. Since values of *D*/*v* are 3.4 and 3.0 cm for molecules I and II, respectively, it is expected that separation efficiency will be higher for molecule II (see Methods for values of *D*).

In conclusion, we have shown that the strength of the molecular propeller effect is underappreciated and can indeed enable separation of chiral molecules into chiraly enriched states within hours when exposed to REFs. The only essential requirement for the method is that the molecule has a non-zero dipole moment, which is likely for most chiral molecules of interest in pharmaceutical industry. All relevant factors such as value of rotational–translation coupling value (*L*_rev_), solubility in non-polar solvents and dipole moment magnitude can be optimized by temporary derivatization with suitable chemical groups. The fields of drug discovery, development and manufacturing increasingly require molecules that are enantiopure. The molecular propeller effect and the technique described herein set the stage for future development of an entirely new method with advantages for both separation (for example, enrichment of enantiomers) and, in particular, the analysis (for example, determination of absolute configuration) of small molecules with less than hundred nanograms of material needed. Furthermore, we hypothesize that the demonstrated molecular propeller effect could be relevant in biological systems to the extent that rotation of molecules in the presence or absence of electric fields may have spontaneously occurred or is occurring in Nature.

## Methods

### Reagents

The racemic 1,1′-Bi-2-naphthol bis(trifluoromethanesulfonate) (MW 550.45 ), the (R)-(−)-1,1′-Bi-2-naphthol bis(trifluoromethanesulfonate) and the (S)-(+)-1,1′-Bi-2-naphthol bis(trifluoromethanesulfonate) were purchased from Sigma-Aldrich and were of highest available purity (97%). The benzene, acetonitrile (both CHROMASOLV type) and the triethylamine (TEA), >99%, were also purchased from Sigma-Aldrich. The R-(+)-2,2′-(1,4-Butylenedioxy)-6,6′-dinitro-1,1′-binaphthalene and the S-(−)-2,2′-(1,4-Butylenedioxy)-6,6′-dinitro-1,1′-binaphthalene (99% enantiomeric purity, MW 430.4) were acquired by custom synthesis contract (by Dr Paramjit Arora, New York University), following the synthesis procedure described previously^26^.

For clarity in the text, 1,1′-Bi-2-naphthol bis(trifluoromethanesulfonate) is referred to as molecule I (Figs 1a and 2d), and 2,2′-(1,4-Butylenedioxy)-6,6′-dinitro-1,1′-binaphthalene is referred to as molecule II (Fig. 4a).

### Simulations and analysis

The optimization of the initial molecular structures and the calculation of dipole moments were performed at the B3LYP/cc-pVTZ level^27^, using the NWChem software package^28^. The electrostatic potential fit of atomic partial charges for molecules I and II was performed using the CHELPG algorithm as implemented in NWChem.

Molecular dynamics simulations were performed at 293 K using the NAMD programme (version 2.9; ref. 29 and CHARMM general force field^30^. The initial structure for the solute and solvent system comprised one solute molecule and 1,463 benzene molecules in a 60 Å × 60 Å × 60 Å periodic box. Simulations were performed on NVT (constant volume) ensemble as implemented in NAMD. The Lennard–Jones potential was switched and truncated from 10 to 12 Å. The particle mesh Ewald^31^ method was employed for calculation of long-range electrostatic interactions. The contributions of Lennard–Jones and particle mesh Ewald to the energy and forces were updated every step. The temperature was held constant by using the Langevin thermostat method with a 5-ps^−1^ coupling constant. A time step of 1 fs was used, and the coordinates were saved every 250 fs. Simulations were performed on a 64-core in-house Linux cluster based on Xeon E5-2690 processors. Each simulation was run for 320 ns. The first 20 ns of each run were intended for equilibration only and were omitted from subsequent analysis. Degree of equilibration was determined by monitoring the values of pressure and temperature of the solvent, and the mean square angular displacement (MSAD) of the solute molecule. Figure 1b shows the MSADs for molecule I calculated along three relevant molecular rotation axes (Fig. 1a); MSADs show the linear dependence that is expected for rotational diffusion. Rotational diffusion constants were determined from the slope of linear fits to the MSAD (Fig. 1b). For comparison, the value of the rotational diffusion coefficient calculated from the Stokes–Einstein formula for rotational diffusion (
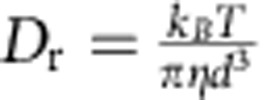
) is ∼3 × 10^12 ^deg^2 ^s^−1^, using *d*=6 Å for the size of the molecule (approximately the size of molecule I for rotation around axis I_1_, Fig. 1a) and benzene viscosity of *η*=0.0065 N s m^−1^; the proximity of the hydrodynamic rotational diffusion value to those obtained from MD simulations (Fig. 1b) supports the applicability of the described approach to study rotational diffusion. The *D*_r_ value of 8.2 × 10^12 ^deg^2 ^s^−1^ for rotation around the relevant I_1_ axis for molecule II was obtained from MD according to the identical procedure as for molecule I (note that it is larger, consistent with the smaller size of molecule II). Note that *D*_r_ values around the three different principal molecular axes are similar (Fig. 1b); therefore, for simplicity of the analysis we only use the value of *D*_r_ around the relevant propeller axis I_1_.

### Calculation of the responding fraction

At equilibrium, the angular probability function of molecular dipole orientations is 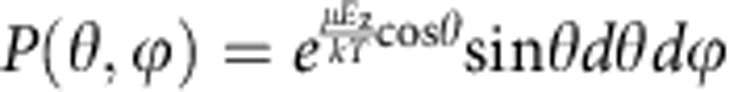, where we assumed that electric field is aligned along the *z* axis (see Supplementary Fig. 1). Axial symmetry leads to the following integrated normalized molecular concentration density profile:


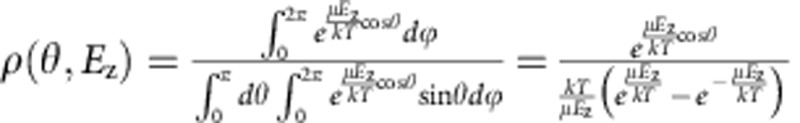
, which is equivalent to equation (2) when the electric field vector is aligned along the *z* axis. The minimum in the dipole density is at *θ*=*π*, that is, when the dipoles are antiparallel to the electric field: 
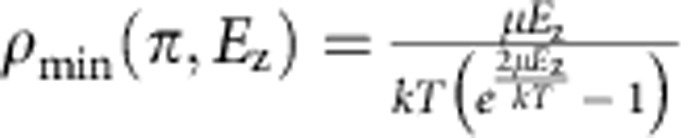
. The total number of non-responding dipoles is then 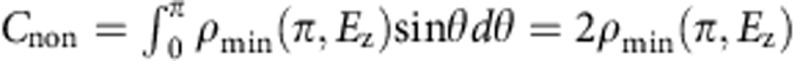 (these are the dipoles below the dashed line in Fig. 1f that on average do not rotate in the rotation direction of REF). The total number of dipoles, 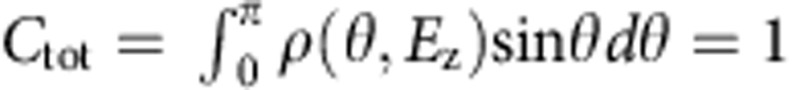 due to normalization. Therefore, the responding fraction is 
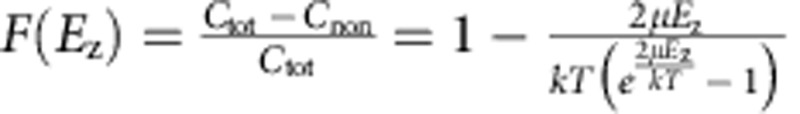
 (the subscript z can be dropped since the formula is valid for any orientation of the electric field). The responding fraction is an average fraction of the molecules that rotate following the REF.

### Effective rotational frequency

The equation (1) is easily solvable analytically at equilibrium but not in the general, time-dependent case. To determine the effective rotational frequency of the molecule at a given rotation frequency of the external REF, we have solved the overdamped rotational motion equation with one degree of freedom that describes deterministic motion of a macroscopic dipole:


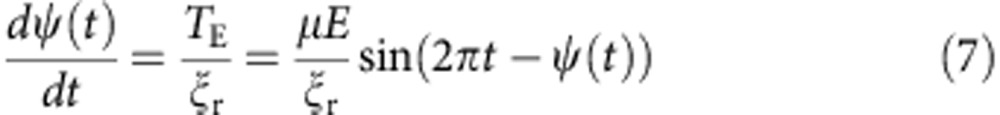


where *T*_E_ is a torque imposed on the electric dipole moment by the field, 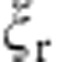 is the rotational friction coefficient determined from the Einstein relation 
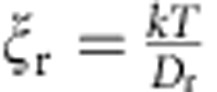
 and *ψ(t)* is the polar angle describing orientation of the dipole moment vector in the *zy* plane (see Supplementary Fig. 1). The term 2*πt*−*ψ*(*t*) in equation (7) represents the phase lag between the REF and the dipole moment. Equation (7) can be easily integrated analytically


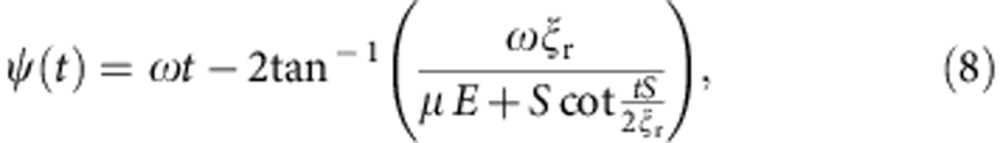


where 
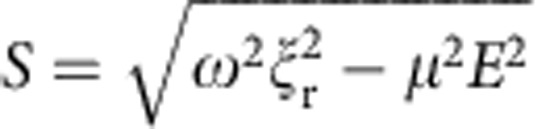
and *ω*=2*πν*. Supplementary Fig. 2a shows the time dependence of the total dipole rotation angle (that is, *ψ(t)*) at three different electric field magnitudes. These data illustrate that above a certain electric field strength the molecule is capable of rotating with the REF, while at lower field magnitudes it ‘slips,' leading to an effectively lower molecular rotation frequency. As illustrated in Supplementary Fig. 2a, the effective molecular rotation frequency is calculated as 
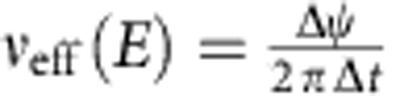
.

Supplementary Fig. 2b shows *ν*_eff_ as a function of the REF frequency *ν*; these results indicate that below 
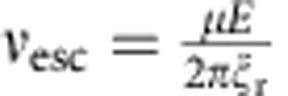
 (for example, *ν*_esc_=0.507 MHz for molecule I and *ν*_esc_=2.1 MHz for Molecule II) the *ν*_eff_=*ν* (and it does not depend on *E*), while at higher field rotation frequencies the *ν*_eff_ falls off rapidly because of ‘slippage' effect, that is, the torque due to dipole–electric field interaction is not strong enough to rotate the molecule at the REF frequency. For example, at the field rotation frequency used in our experimental set-up (0.9 MHz) the effective rotational frequency for molecule I is only 156 kHz, while for molecule II *ν*_eff_=*ν* (that is, 0.9 MHz), mainly because the dipole moment of molecule II is significantly larger.

By taking the time derivative of equation (8) and assuming that field rotation frequency is much larger than *ν*_esc_, one can obtain the following asymptotic analytical expression:


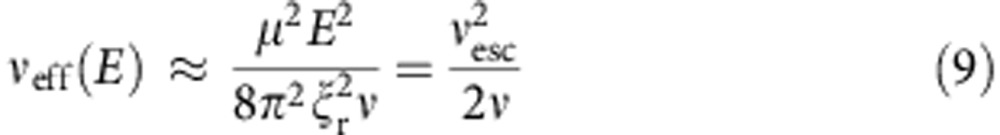


which indicates that when *ν*≫*ν*_esc_, *ν*_eff_ is inversely dependent on the field rotation frequency and proportional to the dipole–field interaction torque squared.

### Calculation of angular correction factor *A*_cor_

For weak electric fields the dipoles diffuse out of the *zy* plane (see Supplementary Fig. 1 for definition of the angles). The electric field torque still causes these molecules to rotate around the *x* axis; however, the molecular propeller axis with highest rotational–translational coupling (I_1_, see Fig. 1a for orientations of principal axes) is not always aligned along the *x* axis; hence, the propulsion efficiency is reduced by a certain factor that we call *A*_cor_. *A*_cor_ is then simply given by a normalized average projection value of the relevant propeller axis (I_1_) on the *x* axis (in the positive or negative direction):


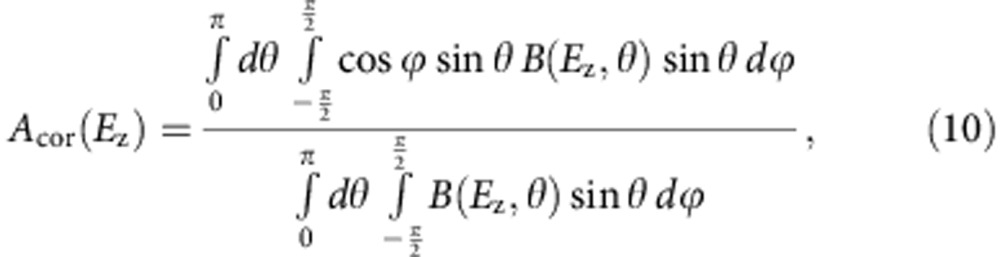


where 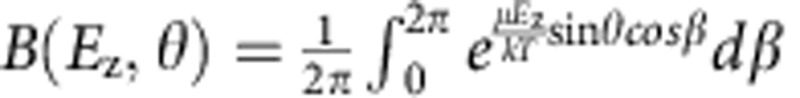.

The *B*(*E*_z_,*θ*) is a mean value of the Boltzmann factor averaged over all possible orientations of the dipole moment around axis I_1_ at any given orientation of the I_1_ (*β* is an integration variable introduced to perform rotation of the dipole moment around the I_1_ axis); *B*(*E*_z_,*θ*) accounts for progressively more likely alignment of the propeller axis (I_1_) in the plane *xy* at stronger electric fields (at stronger fields the dipole moment tends to align more along the *z* axis; therefore automatically, the axis I_1_ that is perpendicular to the dipole moment gets more parallel to the plane *xy*). When 
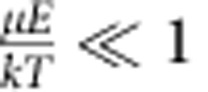
 (0.004 for Molecule II), orientations of molecular dipoles are random, angular distribution function is nearly flat (see Fig. 1f) and *A*_cor_≈0.5 (this approximation is equivalent to setting *B*(*E*_z_,*θ*)=1, in which case *A*_cor_ is exactly 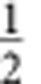). When 
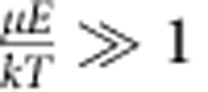
, *A*_cor_(*E*_z_) approaches the value of 0.637. This can be rationalized by noting that when the electric field is very strong, the dipole moment (which is parallel to I_2_) is always in the *zy* plane (parallel to the *z* axis at the time moment shown in Supplementary Fig. 1). *A*_cor_ can then be simply calculated by averaging over the random orientations of the I_1_ axis in the plane *xy* since the rotational–translational coupling for rotation around the I_3_ axis is zero (on the basis of MD simulations and symmetry considerations, see above):


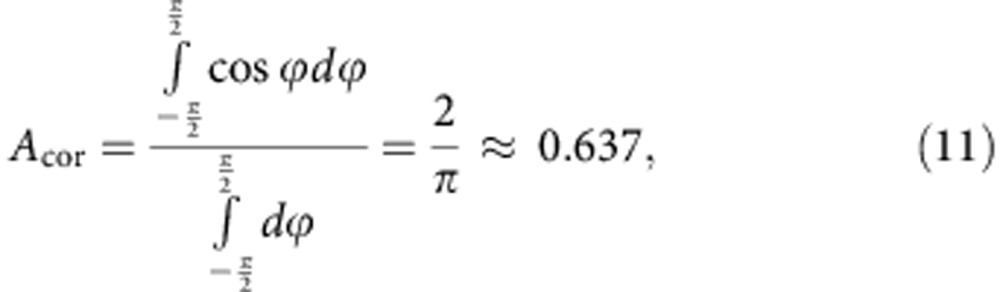


Since the difference between *A*_cor_ values for weak and strong fields is small, we will ignore the dependence of *A*_cor_ on the electric field magnitude and use the low field value of 0.5 (at the field magnitude used in this paper the value of *A*_cor_=0.5000003 for Molecule II).

### Separation chamber and REF

The fluidic path of the separation chamber consisted of a polyimide-coated fused silica capillary (193 μm outer diameter (OD), 75 μm inner diameter (ID), Polymicro Technologies). A 10-cm-long segment of this capillary was surrounded by eight strands of custom-manufactured 41-gauge copper mag wire (MWS Wire Industries, ‘double-quad' polyimide coating; Fig. 2a). The assembly was inserted into a heavy-walled borosilicate glass capillary, or ‘sleeve' (6.4 mm OD, 455 μm ID, VitroCom, length=100 mm). The assembly within the borosilicate glass sleeve was encapsulated using polyurethane with a high-dielectric strength (Hysol US1750). Axially symmetric cooling fins were attached to the outside of the sleeve (100 mm × 19 mm × 2 mm, CoolPolymers thermally conductive plastic) with thermally conductive epoxy (3 M).

The eight wires that extended from both ends of the chamber were connected in adjacent pairs, to create the four electrode poles. At both ends of the chamber, these electrodes were connected to the circuit board that generated the REF within the chamber. The circuit board for the REF generation was custom-manufactured by contract (Trommler Designs, The Product Design Company); it supplied the square-like waveforms to each chamber electrode at 900 kHz and 1,100 V (Fig. 2c). The circuit board design employed precise timing to the gates of high-voltage MOSFETs, to control the four phases (A–D) of the REF (Fig. 2b). The REF generator was powered by a custom 2-kW-high-voltage power supply (Shekonic E&M Co. Ltd). Each waveform was phase-shifted by *π*/2 in a CW manner from electrodes A to D. With a 50% duty cycle, this scheme ensured that there was ∼*π*/4 overlap between each adjacent phase's high (on) states (Fig. 2c). The REF could also be applied in a counterclockwise manner to rotate from D to A. The CW direction of the REF was defined by looking along the direction of sample flow, that is, from the injection to collection stages (for example, from right to left in Fig. 2d).

The theoretical magnitude of the electric field within the separation chamber was determined using the COMSOL Multiphysics software, taking into account the materials and parameters described above. The electric field was 0.6 × 10^6 ^V m^−1^ in the centre of the microfluidic chamber with a gradual decrease of 4% towards the inner walls of the chamber.

The REF strength was limited by both thermal management considerations of MOSFETs on the REF generator circuit board and dielectric characteristics of the chamber materials. The heavy-walled sleeve and cooling fins of the chamber ensured efficient heat transport away from the centre of the chamber. Additional cooling was provided by a fan blowing cold air across the chamber and fins. The chamber temperature was continuously monitored and was ∼16±2 °C; there was only ∼4 °C rise in chamber temperature during operation. In addition, all microfluidic paths were held in exactly horizontal orientation to prevent any potential convection flows.

### Microfluidics control

The experimental set-up consisted of sample injection, enantiomeric separation, in-line chromatographic detection and finally sample collection stages. These stages were implemented around a primary microfluidics line, which was a single continuous capillary to prevent sample profile broadening from microfluidic unions. The entire process was automated and controlled remotely using a Windows PC with NI LabView 2012. After sample collection, the analysis was carried out using a separate CD detector to confirm the efficiency of enantiomeric separation.

The sample (∼6 nl) was injected into the primary microfluidics pathway (polyimide-coated fused silica capillary, ID=75 μm, Polymicro Technologies) using a custom-modified HPLC injection valve (VICI Valco Instruments, Cheminert, with microelectric actuator). The eluent was benzene with 100 μM TEA (to prevent tailing) and the sample concentration was 8–24 mM. Benzene was chosen as the eluent because of its low dielectric constant (∼2.3), which was advantageous to maintain a stronger electric field within the microfluidic path (the electric field magnitude inside separation chamber is proportional to the ratio of dielectric constants of chamber material (fused silica) to the solvent).

After injection, the sample was pumped to the centre of the separation chamber at a flow rate of 0.050 μl min^−1^ (KD Scientific 210 syringe pump). Once the sample was positioned, pumping was stopped and the microfluidics pathway was isolated by closing both the injection and collection valves (the collection valve was a custom-modified VICI Valco Instruments Cheminert 4-port selector valve with microelectric actuator). The REF was then applied to the chamber for any length of time. The centre position of the sample slug within the separation chamber could be controlled with an accuracy of about ±15 mm over time periods of up to 1 week. However, for time periods of more than ∼2 days, the slug spreads diffusively to the point where it starts exceeding the length of the active area of the chamber (10 cm).

After the REF was turned off, both the injection and collection valves were again opened and the pumping was resumed. At this time, the in-line ultraviolet absorbance detector (Paraytec ActiPix D100) was turned on to observe the sample as it eluted from the chamber and then towards the collection valve. The detection wavelength of 300 nm was chosen for the in-line detector since it is above the cutoff wavelength of benzene and both molecule I and molecule II have appreciable absorbance at this wavelength.

The sample was split as it arrived at the collection valve. Precise timing enabled for both the leading and trailing sides of the sample slug to be collected separately into sample vials for later analysis. By using a single capillary with no junctions, very low pumping speeds and minimizing the distances between the stages of the primary microfluidics line, broadening of the sample slug was minimized.

Immediately after sample collection, the benzene was evaporated from the collection vials at 70 °C and 30 Torr. The samples were then re-dissolved in acetonitrile to obtain simultaneous CD and absorption chromatograms (Jasco, CD-2095 Plus, cell volume 44 μl, optical path length 25 mm). The wavelengths used were 227 and 232 nm for molecule I and molecule II, respectively. The amount of sample recovered from each sample vial was very low; therefore, acquiring the chromatograms at these wavelengths allowed for the most sensitive detection of the CD signal (Supplementary Fig. 3). The cutoff wavelength of benzene is ∼280 nm; therefore, acetonitrile was used for CD measurements. Owing to the very small amount of sample collected from each run (0.048–0.14 nmol) and the subsequent solvent reconstitution step, the recovered amount of material collected from the leading and trailing sides of the sample slug varied from run to run by up to 50%, as manifested in variable absorption peak heights in the chromatograms (Figs 3a and 4b). ee (defined conventionally as 
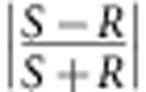
) was calculated on the basis of a calibration curve that was developed from a series of solutions with known ee's. The ratio of the CD signal to the absorbance signal was measured to ensure that this method was concentration-independent.

Ultraviolet/Vis absorption spectra were obtained for samples that had been separated on the instrument and exposed to the REF; these spectra were unchanged as compared with the absorption spectra of either pure enantiomers (Supplementary Fig. 3) or the racemic sample that were not exposed to the REF.

### Lateral diffusion coefficients

The diffusion coefficients, *D*, of molecule I and molecule II were determined experimentally by quantifying diffusive broadening of the chromatographic absorption profile from the in-line detector. The solvent was the same eluent as used in all experiments: benzene with 100 μM TEA. Samples were injected into the capillary and then the pumping was stopped for variable time periods between 0 and 21 h, allowing the sample to spread diffusively. After the waiting time was finished, the in-line detector was turned on, the pumping was resumed and absorption profiles were recorded; Supplementary Fig. 4a shows representative chromatographic profiles for molecule I that were used to determine the full widths at half maxima (FWHM). At least three data points per waiting time were recorded. For one-dimensional diffusion and a chromatograph peak with a Gaussian profile, 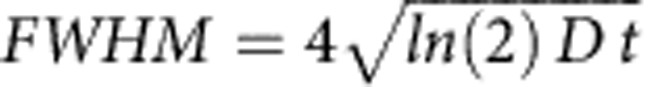, where *t* is the waiting time period. The 
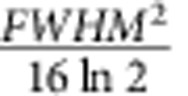
 was plotted versus *t* and linear regression analysis was performed (see Supplementary Fig. 4b). The slope of the linear fit gave the value of the diffusion coefficient for molecule I, *D*=8.3 × 10^−6^±3 × 10^−7 ^cm^2 ^s^−1^ (mean±s.e.m.). A similar procedure for molecule II yielded *D*=1.4 × 10^−5^±5 × 10^−7 ^cm^2 ^s^−1^.

### Statistical analysis

The mean values, s.e.'s and linear regression fits to the ee data (Fig. 4d) and the diffusive spreading data (Supplementary Fig. 4) were performed using Origin 8.1 (OriginLab Corp, Northampton, MA). Linear fits for determination of *L*_rev_ values (Fig. 1c–e) and rotational diffusion coefficients (Fig. 1b) were performed with MATLAB (MathWorks, Natick, MA); s.e.'s of fit parameters were estimated using the block sampling technique^32^.

## Additional information

**How to cite this article:** Clemens, J. B. *et al.* A molecular propeller effect for chiral separation and analysis. *Nat. Commun.* 6:7868 doi: 10.1038/ncomms8868 (2015).

## Supplementary Material

Supplementary Information Supplementary Figures 1-4
