# Erratum: A molecular propeller effect for chiral separation and analysis

**DOI:** 10.1038/ncomms9343

**Published:** 2015-09-18

**Authors:** Jonathon B. Clemens, Osman Kibar, Mirianas Chachisvilis

Nature Communications 6: Article number: 7868 10.1038/ncomms8868 (2015); Published: 07282015; Updated: 09182015

The image in Supplementary Fig. 1 that accompanies this Article was incorrectly rendered while the Supplementary Information file was being prepared for publication. Supplementary Fig. 1 as it should have been published appears below.

## 

## Supplementary Material

Supplementary Information Supplementary Figure 1

