# Supplementary material for: A molecular propeller effect for chiral separation and analysis
Source: Nat Commun. 2015 Jul 28;6:7868. doi: 10.1038/ncomms8868 (PMC4525176; doi:10.1038/ncomms8868)
Supplement: Supplementary Information — Supplementary Figures 1-4 [file ncomms8868-s1.pdf]

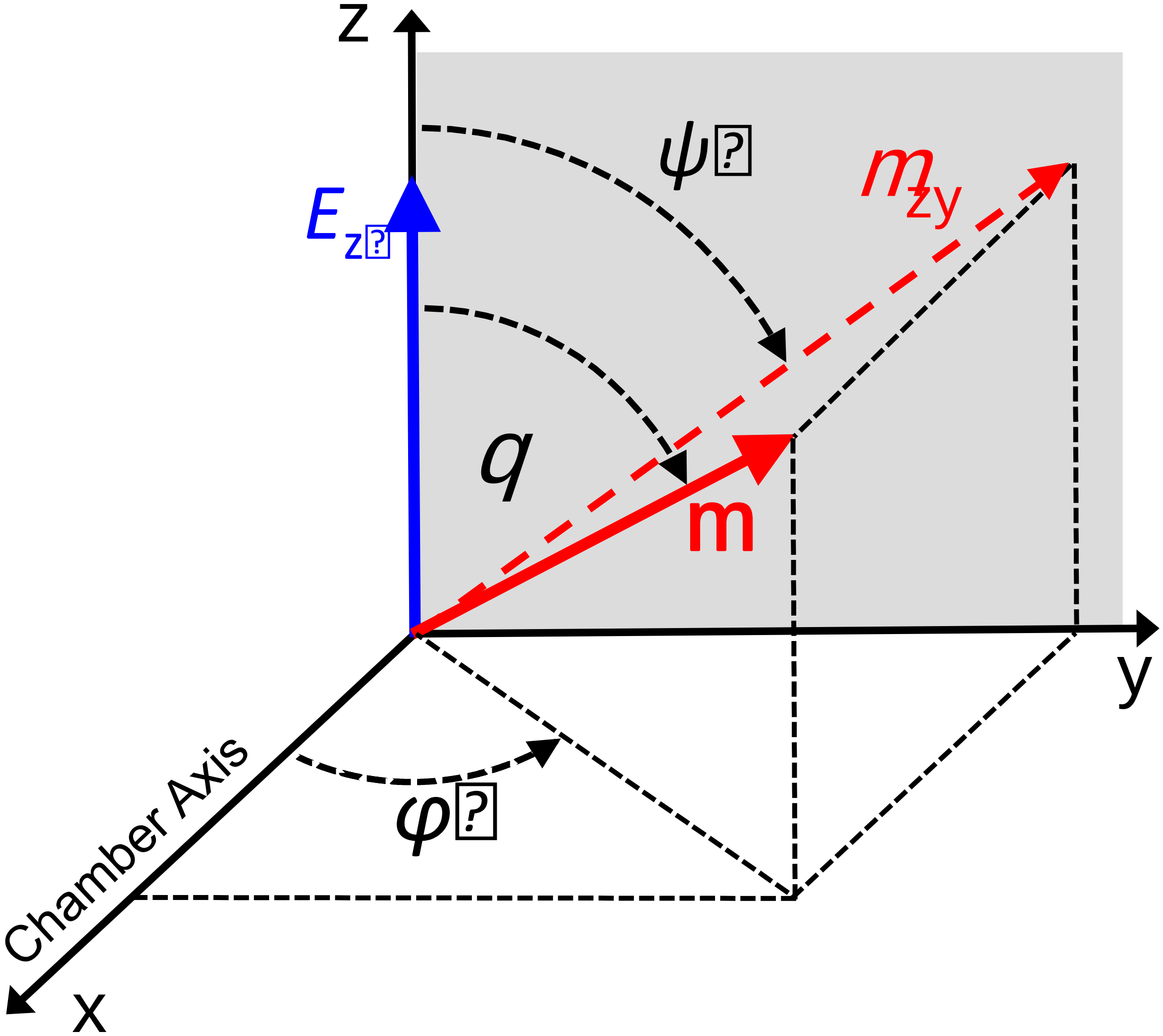

Supplementary Figure 1. Coordinate system.
Definition of angles used to derive equations presented in the text. θ is the azimuthal angle, φ is the polar angle, and ψ is the angle between z axis and any other vector in the zy plane (e.g. the dipole moment projection $\boldsymbol{\mu}_{zy}$ onto the zy plane). Electric field vector is shown at the time instant when it is aligned along z axis i.e. $|\mathbf{E}| = E_z$.

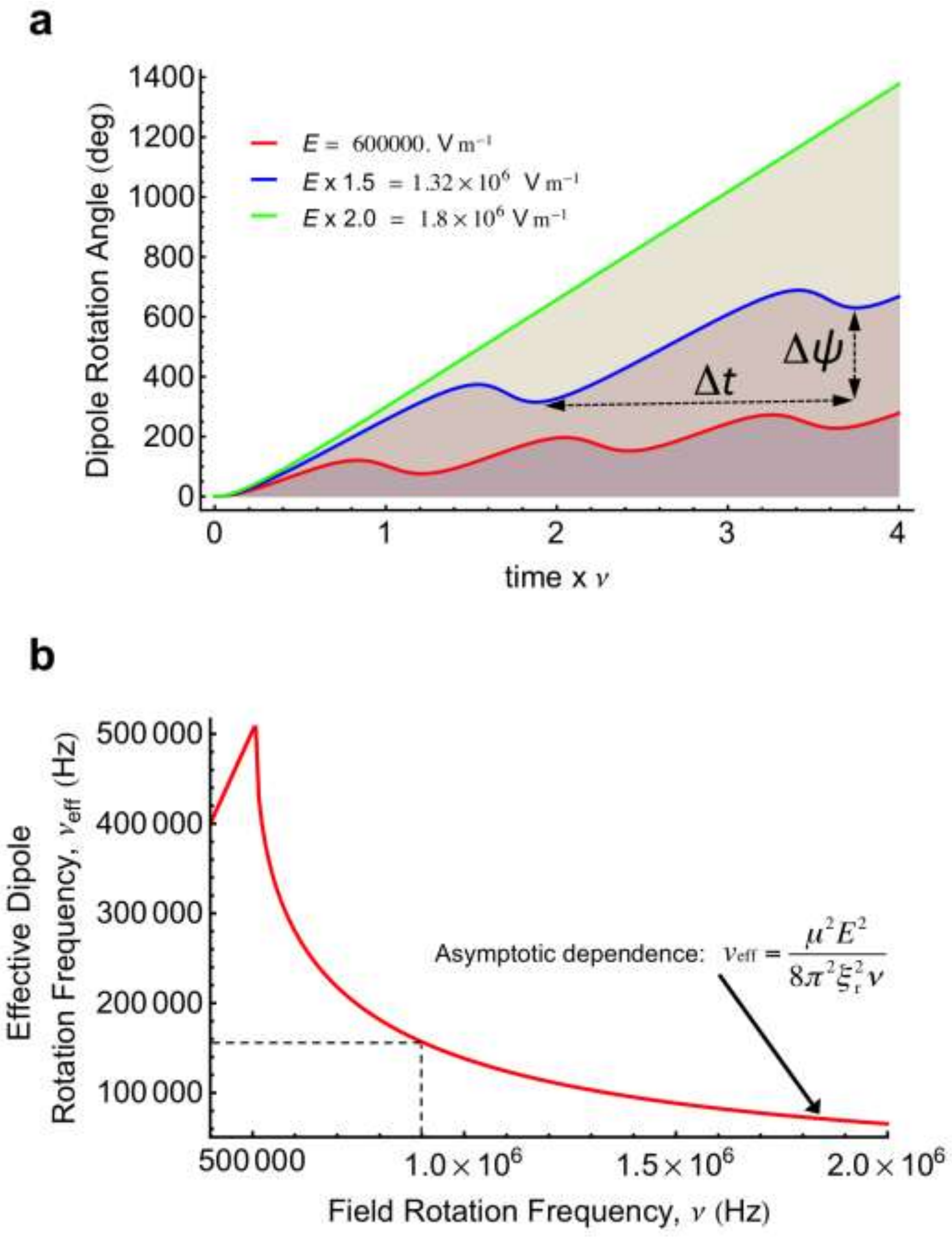

Supplementary Figure 2. Effective Rotational Frequency.
(a) The time dependence of the total molecular dipole rotation angle at three different electric field magnitudes. Parameters used: the REF frequency, ν = 0.9 MHz, rotational diffusion coefficient, D_r = 4 x 10^{12} deg^2 s^{-1}, dipole moment, μ = 5.3 Debye. (b) The effective molecular rotation frequency (ν_{eff}) as a function of the REF frequency, ν. Parameters used: $\nu_{\mathrm{esc}} = \frac{\mu E}{2\pi\xi_{\mathrm{r}}} =$ 0.507 MHz.

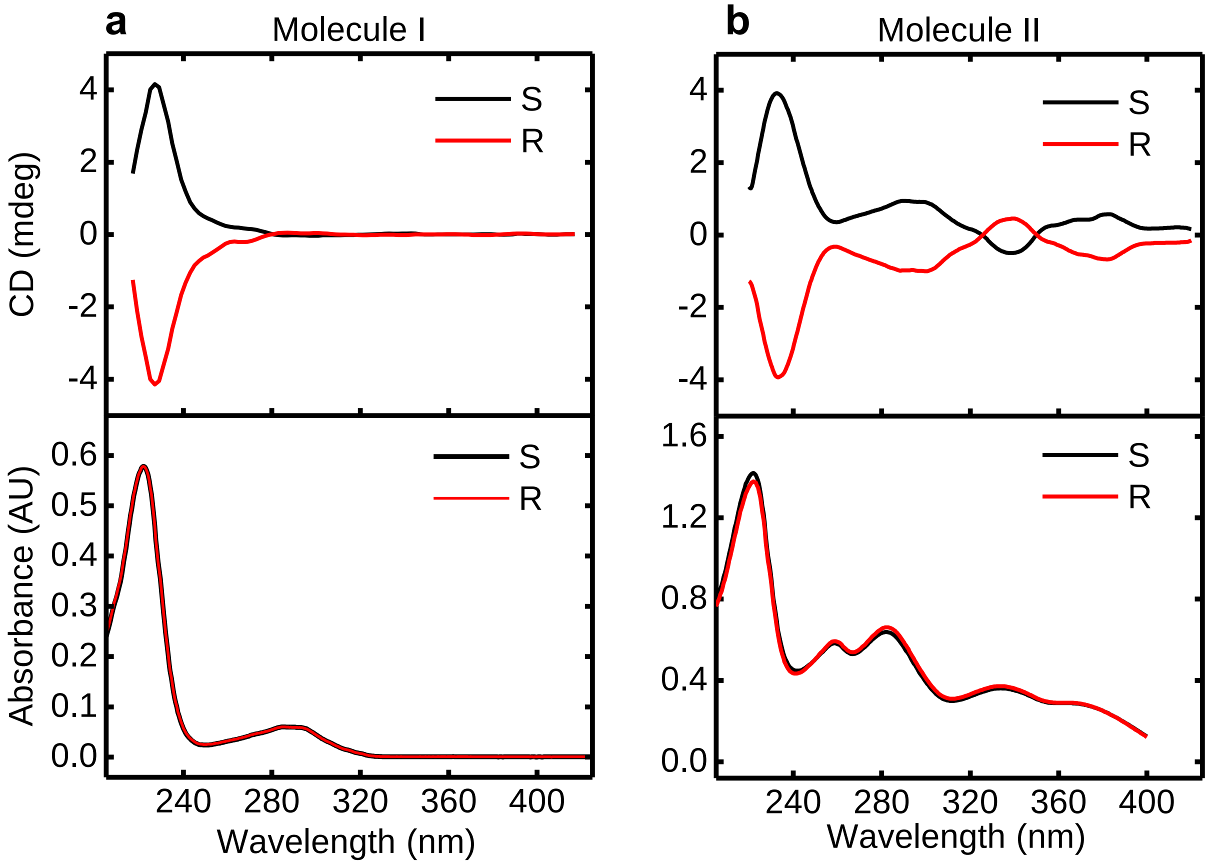

Supplementary Figure 3. Circular Dichroism and Absorption.
Circular dichroism spectra and UV absorbance spectra of the S and R enantiomers of (a) molecule I and (b) molecule II. All spectra measured in acetonitrile as the solvent.

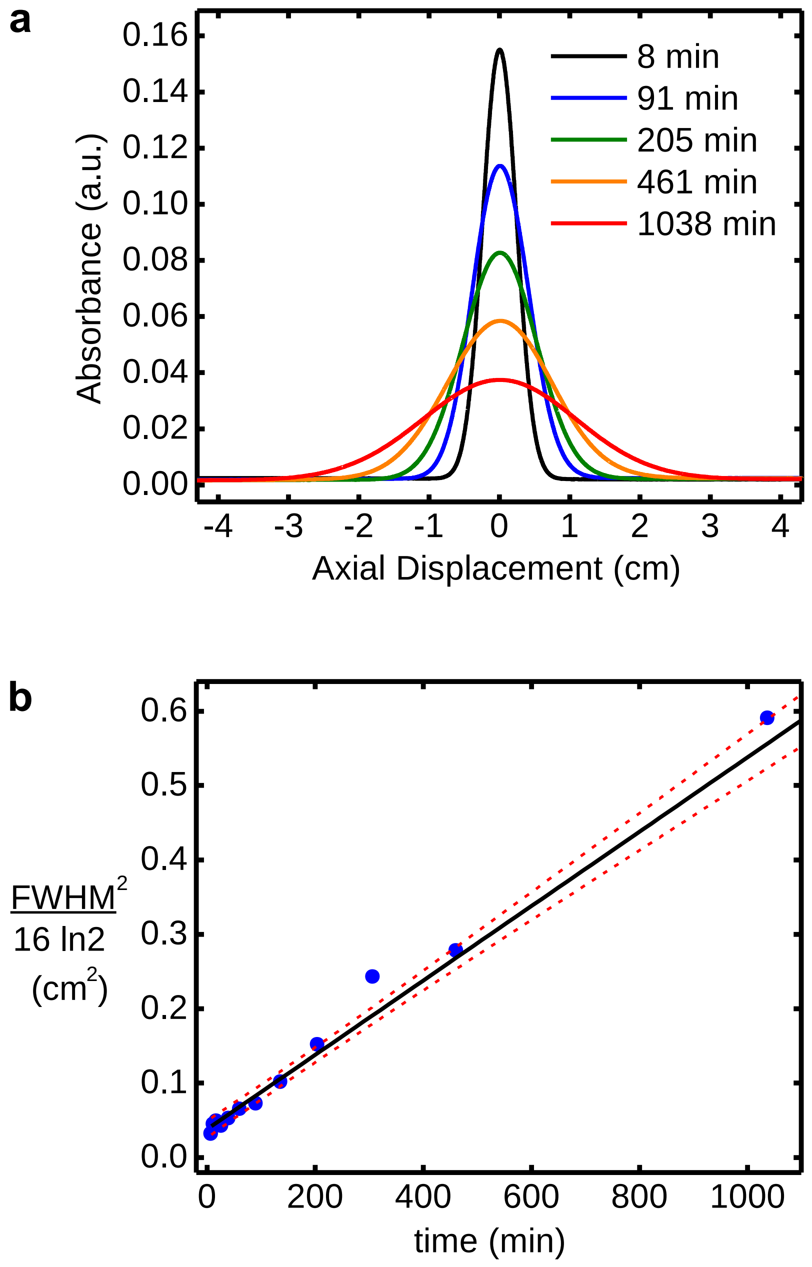

Supplementary Figure 4. Lateral diffusion in the separation chamber.
(a) Chromatographic absorption profiles of molecule I after diffusion within the chamber of the experimental apparatus for specified amount of time in the absence of the REF at ~ 20 °C. (b) Full half-width values of chromatographic absorption profiles as function of time in the absence of the REF; solid black line is a linear regression fit yielding the diffusion coefficient of molecule I, $D = 8.3\times10^{-6} \pm 3\times10^{-7}$ cm^2 s^{-1} (mean ± s.e.m.). Dashed red lines are the upper and lower 95% confidence intervals of the linear regression fit. Data are from the chromatographic absorption profiles recorded using the in-line detector. Each data point is an average of 3 measurements. The solvent was benzene with 100 μM TEA at ~20 °C.
