# Supplementary material for: Erratum: A molecular propeller effect for chiral separation and analysis
Source: Nat Commun. 2015 Sep 18;6:8343. doi: 10.1038/ncomms9343 (PMC4595713; doi:10.1038/ncomms9343)
Supplement: Supplementary file 1 [file ncomms9343-s1.pdf]

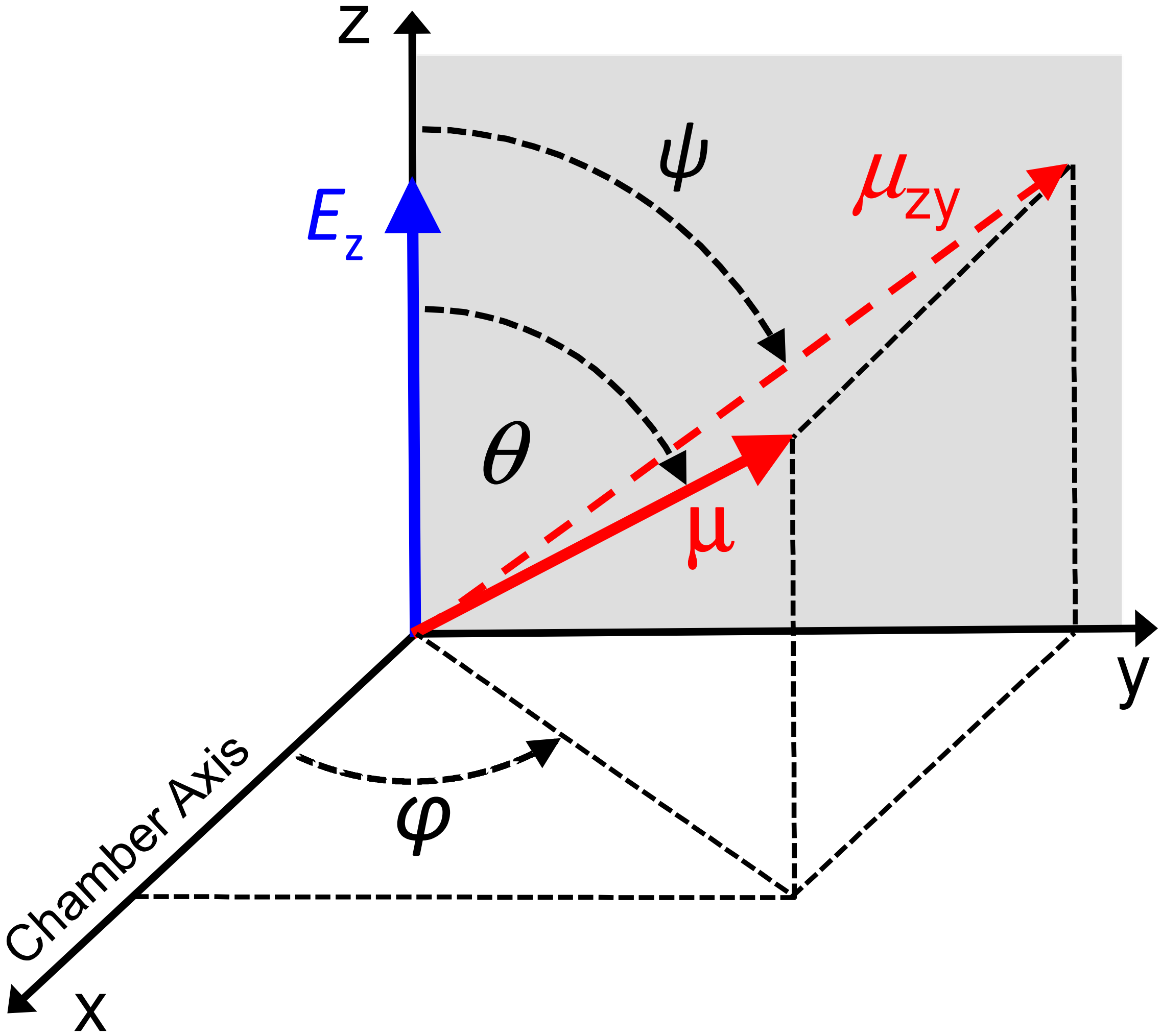

Supplementary Figure 1. Coordinate system.
Definition of angles used to derive equations presented in the text. θ is the azimuthal angle, φ is the polar angle, and ψ is the angle between z axis and any other vector in the zy plane (e.g. the dipole moment projection $\boldsymbol{\mu}_{zy}$ onto the zy plane). Electric field vector is shown at the time instant when it is aligned along z axis i.e. $|\mathbf{E}| = E_z$.
